# M. Goundret on a New Mode of Curing Cataract

**Published:** 1826-07

**Authors:** 


### Observations on Cataract.*

That morbid change in the organ of sight, which consists in opacity of the crystalline lens or its capsule, and to which the name of cataract has been applied, belongs, both by its situation and by the causes which give rise to it, to the class of internal disorders. The remarkable want of success, which has hitherto attended all attempts to remove it by medical treatment, has, however, universally transferred the management of it to the hands of the surgeon. It must be acknowledged, that the operations for the cure of this affection have, of late, attained a high degree of perfection, and that a proportionate number of striking cures has been the result. It could be wished, notwithstanding, that a combined practice might be directed against this malady, which frequently becomes the cause of one of the most trying infirmities of age, and occasionally is met with in the earliest periods of life.

The author, in treating epilepsy and mania by cauterization of the head, had had occasion to notice a remarkable improvement in amaurosis and cataract, in cases in which they happened to co-exist with the affection, which formed the principal object of attention. The observation appeared perfectly admissible, for there is no more difficulty in conceiving the removal of the physical cause of amaurosis or cataract, than that of mania or epilepsy.

The result of the author's experience with respect to amaurosis, was laid before the Institute in a former paper, and the attention which that received encouraged him in the prosecution of the researches detailed in the communication now before us.

In seeking to apply to cataract the treatment which he had adopted in chronic affections of the head, and in gutta serena, Dr. Gondret soon found that his facilities for experiment were much less considerable. The cases of amaurosis which presented themselves were numerous, because this affection is frequently abandoned as hopeless, but operation being the generally received method of treatment in cataract, it was not till after long waiting, that a few almost accidental opportunities occurred for the trial of a practice, which he conceived equally applicable to this, as to other affections of the eyes or brain.

He carefully noticed the facts as they occurred, and waited until he had accumulated a number of analogous results, before he considered himself warranted in drawing any thing like a general conclusion.

The seat of the derangement was particularly favourable to these researches. The existence of cataract becomes obvious long before it has arrived at an advanced stage, and it is well known, that surgeons generally wait to perform the operation for its cure, until the opacity of the lens and loss of vision are complete. Other internal affections are materially different. A direct examination cannot be made, and the diag-

---

* Being the subject of a Paper read to the French Institute, by L. F. Gondret, M.D.

nosis is necessarily less positive, being rather the result of induction than of the immediate evidence of the senses.

As a guard against the possibility of his too hasty admission of facts in support of his own views, Dr. Gondret made his observations in conjunction with several of his enlightened professional brethren, who would not have failed to warn him, had he been falling into error.

He took particular care not to be misled, by mistaking an apparent for a real cure, as is often unavoidably the case, where the practitioner too soon loses sight of his patient. He saw the necessity of continuing the observations as long as possible, and did so for months, and even years.

1*st Case*. M. Pepin, aged 59, of a good constitution, but of rather a nervous temperament.

After a forced march, in the year 1816, he was attacked with severe fever, accompanied by intense head-ache and delirium, and followed by a general swelling of the limbs. During his convalescence, he perceived, for the first time, a black speck, which seemed to dance between his right eye and the objects which he looked at. Six months after, a cloudiness, the precursor of cataract, presented itself in the left eye. In the winter of 1819-20, he experienced, during the space of a month, the most exquisite pain in the right temple: this was succeeded by inflammation of the left eye, which was relieved by leeches. In January 1822, the pains in the right temple returned with great severity, but ceased by the use of an opiate pill. In April, Pepin consulted Dr. Gondret. At this time, he could see nothing with the left eye, in which the cataract had now existed four years. A grey spot in the centre of the crystalline lens indicated the commencement of cataract in the right eye also. He was unable to read, for more than a short time, without fatigue. The floating spots which he perceived with the right eye were become more numerous, the pupils were rather dilated, and the irides almost fixed.

Dr. G. considers, that the malady, with which Pepin had been attacked six years previously, as already mentioned, was the principal cause of the derangement of the visual organs:—that the brain, in the first instance, becoming affected, an extension took place, producing—

1st. Numerous filaments, and approach to opacity of the crystalline, with feebleness of vision in the right eye.
2ndly. Complete cataract of the left eye.
3rdly. Pains in the right temple.
4thly. Inflammation of the right eye.

Continued application at the desk had, doubtless, favoured the progress of the complaint.

Guided by experience of the excellent effects of a local treatment in some of the several affections of the brain, even when congenital, Dr. G. was induced to propose to his patient cauterization of the sinciput, as offering the most favourable chance of relief. He hoped to arrest the progress of the cataract in the right eye, and to render the parts more susceptible to the influence of other agents which, whether local or general, might favour the restoration of the organ to its natural state.

On the 4th of April, the cauterization was performed with ammoniacal pomade.

On the 22d of May, the cloud which had seemed to cover the right crystalline was become less, apparent vision was a little improved, but filaments still remained. An electric current, produced by a voltaic trough of thirty plates, was passed between the right supra-orbital nerve and the right eye. An immediate, but momentary, improvement of sight was the consequence; the nerves, however, continued to feel the shock till the following day.

June 1st. The right eye was perfectly clear, and vision increasingly strong.

July. The cloudiness of the right crystalline was returning, and vision was a little more obscure. The ulceration at the sinciput was merely superficial, and was reduced to scarcely the third of an inch in diameter. It was then extended and made deeper by another application of the pomade.

August. No opacity of the right crystalline. The sight continues constantly good. The cataract in the left eye had changed from a dead white to a grey colour.

June, 1823. The right eye is in its natural state, and vision is good. The left crystalline approaches more nearly to a greyish black, but that eye merely perceives the presence of light.

August, 1825. The cure is permanent with respect to the right eye. The cataract in the left is scarcely visible, but vision on that side has made no progress.

*2d Case.* John James Henriot, aged 71, affected with cataract in both eyes—the sight of the left eye gone; the cataract in the right less advanced, but very obvious; the sight of this eye sensibly impaired for several months.

October, 1822. The sinciput was cauterized with a red hot copper instrument, the patient having chosen the most painful, but at the same time the most expeditious method.

The sore was kept open four months, during which time an ammoniacal collyrium and galvanism were also employed: the bowels were kept open as far as possible by regimen, but when this failed, laxatives were made use of.

The opacity of the right crystalline gradually disappeared, and the sight of that eye became completely restored. The opacity of the left lens diminished, and there was some return of sight, but the improvement on this side was not permanent.

August, 1825. The patient has had no return of cataract in the right eye, with which he continues to see well.

*3d Case.* The Countess of Monchenue, aged 80, had impaired sight in common with other infirmities attendant on her advanced period of life. On the 11th January, 1823, on waking she discovered that she was blind; in the course of the day there was some return of sight in the right eye, but none in the left.

On the 27th, this lady consulted Dr. G. who found the conjunctiva and cornea dull in both eyes. The pupils were very much contracted, and there appeared to be some lenticular opacity. She could see only with the right eye, and that but at a very short distance. She could neither read nor write, distinguished colours but in a very confused manner, and imagined that she saw variegated flowers on a gown which was perfectly white. An oculist, whom she had consulted, recommended an operation, when the right eye should have become opaque as well as the left.

Dr. G. did not consider this case either complete cataract or amaurosis, but believed that the derangement was more general, each part of the organ bearing its share.

The case was recent, and not attributed either to apoplexy or cerebral plethora, both of which affections Dr. G. regards as leading to morbid changes in the eye, which, even when acute, are extremely difficult of cure.

The Countess readily consented to cauterization, which was effected by means of the ammoniacal pomade.

This constituted the principal part of the treatment, but other means were also had recourse to. Cupping-glasses were moderately applied to the neck: an ammoniacal collyrium and galvanism were made use of, and some laxative medicines given. This plan was followed up for four or five months.

After the first month, sight was restored in the left eye and improved in the right. The dulness of the conjunctiva, cornea, and anterior chamber, had disappeared from both eyes, and they had resumed a certain degree of brightness.

Towards the close of the treatment, vision was so far restored that the patient could discover the colours of objects, and could even read and write.

At the time the author was writing, this state of sight had continued for two years, although, as is worthy of remark, the other faculties had, in the mean time, been materially declining.

In this case there appears to have been imperfect cataract, complicated with opacity of the membranes, situated in the anterior of the globe, and with an impaired state of the nerves of the eye.

*4th Case.* Mrs. M. having large eyes and myopism from her infancy, had been for six months affected with amaurosis, which came on after measles, which she had when about six months advanced in pregnancy. Three months after delivery, she consulted Dr. G. who, at the time, observed no apparent organic change. The right eye was rather larger and more prominent than the left. The pupils were perfectly mobile. This patient had always been subject to ophthalmia. She was unable either to read or write, and being myopic, could not easily direct her steps. Cauterization of the sinciput, cupping, the ammoniacal collyrium and electricity restored the sight of both eyes, though less completely that of the left, which had long been weaker than the right. Mrs. M. seemed, for some time, to have even stronger sight than she had ever before possessed. As the lady had again become pregnant, Dr. G. thought it expedient not to continue the treatment longer than four or five months. In the spring of 1822, she set out for Switzerland, where she intended to be confined. The improved state of sight continued, though with occasional relapses, which were quickly relieved by the ammoniated collyrium and electricity. A short time after her accouchement, by exposing herself to wet and cold, she brought on ophthalmia, and vision was again impaired. A month after, she returned to Paris, and at that time was unable to see with the left eye, in which there was an obvious cataract—there was no trace of the kind in the right, though vision was much impaired on that side also.

Two months later, the treatment at first employed was again had recourse to—the sight of the right eye was restored, and this improvement has now continued permanent for two years, but, on the left, the cataract remains, and there is no return of vision.

The cataract was probably the result of the inflammation set up in the eye, but the patient's absence in Switzerland prevented Dr. G. from observing the relation which these two affections bore to each other. In less than two months the opacity of the crystalline and blindness were complete, and a course of treatment, persisted in for two years, effected no sensible change in that eye.

This case forms a striking contrast with some of the others which are related, particularly with that of Pepin.—In him, vision was deteriorated in the left eye, nearly three years before cataract commenced. Under the course of treatment, the lens changed its distinct white for an almost invisible grey; yet, the restoration of sight was confined to the mere perception of light, doubtless on account of the irremediable nature of the older affection.

Nearly a similar instance occurred in the case of M. Delcros. The patient had injured his eyes by application to books. His sight, already poor, became considerably weaker, in consequence of attacks of inflammation affecting the head and eyes. Symptoms of amaurosis and cataract led to the adoption of the mode of treatment employed in the former cases. The cautery of the sinciput produced a temporary diminution of the opacity of the crystalline; but the malady again resumed its progress, and blindness became complete.

*5th Case.* The Baroness A. of a vigorous constitution, and subject, from her youth, to an eruption on her face, consulted Dr. G. in June, 1823. She had, at that time, two well-marked cataracts, which were considered to depend on the opacity of the capsule of the lens. Regarding this as a case already too far advanced to give way to his plan of treatment, the doctor recommended an operation. To this the lady was much opposed, and resolved to bear with loss of sight rather than submit to it. Under these circumstances, Dr. G. consented to make the experiment. The sinciput was cauterized with ammonia, but the Baroness constantly refused to be cupped, though the plethoric state of the head seemed frequently to demand the adoption of this measure. Vision, notwithstanding, had, in less than a month, begun to improve. At the same time, the opacity of the crystallines was manifestly diminished—the white below was changed for grey. Galvanism and the ammoniacal collyrium were then added to the former treatment. On either of these means being employed, the sight became clearer and stronger, and continued in that state through much of the succeeding part of the day. The patient frequently stated that she could see her way better, that she could distinguish persons more easily, and could even read, provided that the book was printed in a large type.

This state of things continued upwards of a year.

Towards the month of September, 18 months from the commencement of the treatment, symptoms of plethora shewed themselves about the head and face, but the patient, as before, was unwilling to submit to cupping, and leeches, applied to the anus, produced only partial relief. From that time dimness of sight continued to increase in the right eye. It had, previously, always been the weaker, and its crystalline had presented a greater degree of opacity—whiteness returned, and in the course of a few days rapidly increased.

In November, the left eye lost ground, and its lens became more opaque than it had been since the commencement of the treatment, which was thenceforth discontinued. She was advised to have recourse to operation. This was subsequently performed on the right eye by Roux, and was attended with complete success.

*6th Case.* The Princess of Broglie Revel, aged 60, had been affected for nearly 20 years with very severe head-aches.

The cephalalgia was frequently accompanied by vertigo, giddiness, and at times by an instantaneous loss of sensibility.

For several months she had found her sight obstructed by a sort of mist, which partially concealed the colour and size of objects—she was able to read and write, though with some difficulty, and but for a short time together.

There were traces of commencing cataract; but, in other respects, the eyes appeared sound.

Cauterization was proposed, as adapted both to the cataract and to the cephalalgia.

After eight or ten days' treatment, the head-ache and mist were removed—and opacity had disappeared from the lens.

Dr. G. then discovered a slight silvery whiteness deeply seated in the eye; he attributed it to some change which age had effected in the choroid—there was no glaucoma.

The princess, relying too much on her strength of constitution, in spite of the caution which she received, neglected to defend herself sufficiently against cold and moisture.

Early in October, the Princess exposed herself in the rain. An eruption appeared around the cauterized spot, and extended to the forehead, and did not leave the patient for three weeks.*

After a month's confinement to the house, this lady went on foot, on an unfavourable day, to pay a visit to Dr. G.

The effect of this exposure was highly injurious to her. Spasmodic movements of the limbs soon came on, sensation became perverted, and her tongue, without offering any change in its appearance, gave her the sensation of being dry and rough like a rasp. Every thing which she touched felt to her like parchment.

It should be remarked, that the Princess had always been subject to nervous affections.

A consultation was proposed by Dr. G. and Portal, Laennec, and Hervez de Chégoin, were called to see her in conjunction with him. The two latter, influenced by the account which the patient herself gave of the benefit which she had derived from the cautery, and conceiving that the existing symptoms were to be attributed to an affection of the spinal marrow, proposed that from time to time similar cauterization should be made along the course of the spine. This advice was particularly gratifying to Dr. G. who apprehended that the previous treatment had not been persisted in for a sufficient length of time to confirm the cure of the cataract. Portal did not meet Dr. G. until the evening: he was then disposed to temporise, and the treatment was consequently limited to a weak ammoniacal liniment, in conjunction with anti-spasmodics and bland drinks. Some degree of fever, notwithstanding, came on, and was accompanied by symptoms of gastric irritation, but these were removed by the use of a laxative. The spinal affection increased. In a few days the patient found difficulty in breathing and swallowing, and shortly after, contractions were observable in the extremities. The dyspnœa ceased on the production of vesication at the epigastrium, affected by means of ammonia. The professional attendants united in recommending that a current of electricity should be passed through the lower extremities. Experience having shewn that the epidermis affords some opposition to the introduction of the electric fluid, it was deemed advisable, as a preparatory step, to produce small spots of ulceration behind the head of each fibula and at the ancles. The electric current, introduced at these excoriations, produced an immediate cessation of the contractions. Two days after this single application of electricity, the patient complained of the inconvenience which she experienced from a sort of boil, situated at the upper part of the right thigh. It turned out to be an anthrax, which her physicians hailed as an admirable and salutary effort of nature. A few days dissipated the symptoms which had been two months in establishing themselves. Low diet was changed for the analeptic, and return of strength was further promoted by a visit to the country. Some time after, Dr. G. saw her

* On two other occasions Dr. G. has seen erysipelatous inflammation come on, either in the neighbourhood of the ulcer or on the face, in consequence of similar exposure.

highness, free from head-ache, in excellent health, with her eyes quite well. She undertook a journey to Copenhagen, where she continued to gain flesh and strength.

In August, 1825, the Princess had returned in good health to Paris.

*7th Case.* Madame Leleu, 66 years of age, on the 8th September, 1824, consulted Dr. Gondret on the recommendation of Dr. Dutrembley; Dr. Bessiere saw her at the same time; this lady had two cataracts of a blackish grey colour: she could only distinguish objects at a few feet distance. It seemed to her that she had before her eyes a thick fog, which became less dense towards evening; she could not find her way without difficulty: she described her affection as not being of more than three or four months standing.

The sinciput was cauterized with ammoniacal unguent, and cupping glasses were applied to the neck. In six weeks, the mist before the eyes had become less dense, vision was more distinct, and the cataracts had less opacity.

19th February, 1825. The patient no longer saw the mists of which she had complained, but filaments or specks seemed to play between her eyes and the objects which she was viewing; the cataracts could scarcely be distinguished.

In April, the cataracts were not to be seen, sight was good, but there were some relics of floating filaments.

*8th Case.* Sargent, aged 67 years, formerly a cabriolet driver, affected for seven years with deafness, which he attributed to having slept for several hours in his cabriolet, during a period of intense cold: his sight had failed him for a year previously to his consulting Dr. G. for the first time, on the 7th of October, 1824. The sight of the left eye was gone, the lens was white and very opaque. There was less opacity in the right eye, but vision with it was very weak, and at times wholly wanting. The occasional blindness seemed to depend on the conjunctiva which was in a state of chronic inflammation, attended with but little pain—it was red, and bathed with tears and mucus. The commencement of this ophthalmia had been attended with long-continued and intense head-ache.

8th October. The sinciput was cauterized with ammonia—cupping and ammoniacal collyrium were employed.

25th October. The conjunctivæ were less red and swimming. The sight of the right eye was stronger. That of the left appeared to have undergone a little improvement, and hearing, at momentary intervals, seemed to be rather less dull.

April, 1825. The right eye was free from opacity, and its vision was good, the chronic ophthalmia no longer existed. The opaque matter, in the left lens, appeared to be less homogeneous. The sight, which had been lost, was so far recovered, that Sargent could distinguish features, when seen in profile.

*9th Case.* Bourgenot, aged 50, a serjeant belonging to a corps at Bicêtre was attacked, on the 30th of August, 1824, with inflammation of the right eye, attended with pain, extending from the upper part of the orbit to the centre of the eye, which had totally lost the power of vision: he had also weight and pain of head. He was bled with the lancet, twenty leeches were applied to his neck, a large blister to the arm, and a seton was inserted at the nape of the neck.

On the 8th of December, the commander of the corps referred Bourgenot to Dr. Gondret. The sight of the right eye was quite extinct, the conjunctiva was inflamed and swollen with a sort of chemosis—but free from pain, the pupil was fixed and contracted, and cataract was very obvious: the sight of the left eye was constantly impaired by a thick mist, and was, at times, quite confused—there was an evident degree of opacity of the crystal which was of a grey white colour.

On the 9th of December the seton was suppressed, the sinciput was cauterized, and cupping-glasses were applied to the neck.

17th December. The mist had sensibly diminished, and was, at times, altogether absent: vision was more distinct: the patient, who for a long time had only been able to read with glasses, and but for a short time together, could now read much longer and without glasses.

August, 1825. The cataract of the left eye was almost imperceptible; vision was good on that side. On the right the conjunctiva was cured—the pupil was still contracted, but less fixed. The crystalline remained very visible, though it was less opaque and dense than formerly. The sight of this eye, which had been completely lost, had so far returned that the patient could perceive the countenances of persons, especially when looking out from the smaller angle of the eye, in which direction the crystalline was the least opaque.

*10th Case.* The next case shews the possibility of restoring vision after the operation of cataract has been performed without effect. Dr. G. had already brought it forward in a memoir on the use of actual cautery.

Leroi, a single lady, of 60 years of age, of a good constitution, after having been completely drenched in a storm, in June 1811, observed that her sight greatly failed. By the 24th July, she was completely blind, from the formation of two cataracts: the operation of extraction was performed, but without procuring any restoration of vision.

After seven years of blindness, Leroi consulted Dr. Gondret: she perceived a difference between night and day, but could distinguish neither form nor colour.

Her eyes were bright, the corneæ transparent, and presented, in each, a transverse white cicatrix, but it was the most marked on the right side—the iris was invisible in that eye; in that of the left, instead of a circular pupil, there was a vertical linear aperture, widest in the middle, where it was about half a line in breadth.

Dr. G. advised the lady not to use any treatment, but as she urged that something might, at least, be attempted, to relieve the constant violent head-aches, which aggravated her affliction in the loss of sight, he employed cauterization of the sinciput.

The next day the patient perceived the light better; with the left eye she could even distinguish the face from the rest of the head: with the right eye she merely recognized the presence of light.

On the 25th of June, she could see enough with the left eye to find her way from place to place—the aperture in the iris had become oval—in the right, the iris was still invisible.

17th July. The patient went to the Hotel Dieu; Dupuytren made an artificial pupil in the right eye—the aperture in the iris was of a triangular figure, having its largest angle upwards. From that time she was able to see with both eyes—went about alone, and could write, though she was unable to see the letters.

In August 1825, she could both read and write.

The foregoing are all the cases which Dr. Gondret has given in his Memoir; he could have added many others, but in so doing he would only have been making a needless repetition. Those which he has related, warrant the attempt to oppose cataract by the same means which, on a former occasion, he recommended for the treatment of chronic affections of the brain and eyes. The probabilities of success must vary with the degree and complication of the affection. When the opacity of the lens is strongly marked, and vision is nearly extinct, the time for successful treatment may be gone by; but, as the means proposed have always a tendency to improve the condition of the brain, eyes, and other organs, they may still be had recourse to, when the operation offers little hope of success, or as a preparatory step to that mode of treatment.

Practitioners will probably be the less disposed to reject these observations, when they consider that nature, in some very rare cases, effects a spontaneous cure of cataract.

An example of this kind was communicated to the author of the Memoir by Laennec. Something analogous may be remarked in the case of the Princess de Revel, in whom the spontaneous production of anthrax gave rise to the sudden disappearance of the symptoms dependent on the affection of the spinal cord.

A note is given from Magendie, who remarks that the observations of Dr. Gondret seem to afford an application of his own experiments on the fifth pair of nerves.

Why should we not, says Dr. M. by acting on these nerves, be able to modify the nutrition of the eye which is evidently dependent on the integrity of this pair—and, if the diminished action of these nerves lead to opacity, why should not their excitation lead to the removal of that state? We have nothing certain on this point, but the investigation merits the attention of physiologists and physicians.

We shall not attempt to offer many remarks on the Memoir of Dr. Gondret, which, being short, we have given almost entire. The mode of treatment which it is designed to recommend, is of easy application, and as, with the exception of a temporary sting on the application of the ammoniacal pomade, the inconvenience to the patient is very trifling, few could object to it, were the prospects of benefit less considerable than those which the author of the paper holds out. The number of patients which we have met in the consulting room of Dr. Gondret afforded no slight presumptive evidence in favour of the efficacy of his practice. Its influence was particularly striking in a case of almost congenital amaurosis of long standing, and in other respects of a most unpromising character.

The prescription for the pomade is not given in the Memoir, but Dr. G. stated to us that it was made with very strong liquor ammoniæ, prepared under considerable pressure.

If the still equivocal virtues of acupuncture have any real existence, their influence in ophthalmic affections is probably analogous to that of Dr. Gondret's method.

Amongst the cases at the Hôpital de St. Louis, which were subjected to the examination of the Commission appointed by the Institute to investigate the subject, were several of affections, both of the internal and external parts of the organ of sight. Every remedy but acupuncture being excluded, the improvement was either complete or considerable, yet the treatment and the cure may not have been connected in the relation of cause and effect. The idea that they were so is in some degree supported by Dr. Sarlandrier's pamphlet on Electro Puncture, and on the use of Moxa and Acupuncture amongst the Japanese. It is not only stated that, in the East, the insertion of the needle between the skin and the cranium is recommended in ophthalmic affections, but that Demours, the oculist, has long employed acupuncture of the neck, and that Sarlandrier has adopted the same plan himself.

---

